# Assessment of Optic Nerve Impairment in Patients with Neuromyelitis Optica by MR Diffusion Tensor Imaging

**DOI:** 10.1371/journal.pone.0126574

**Published:** 2015-05-11

**Authors:** Zhiye Chen, Xin Lou, Mengqi Liu, Dehui Huang, Shihui Wei, Shengyuan Yu, Lin Ma

**Affiliations:** 1 Department of Radiology, Chinese PLA General Hospital, Beijing, 100853, China; 2 Department of Neurology, Chinese PLA General Hospital, Beijing, 100853, China; 3 Department of Ophthalmology, Chinese PLA General Hospital, Beijing, 100853, China; Chinese Academy of Sciences, CHINA

## Abstract

**Background:**

Diffusion tensor imaging (DTI) has been used for the evaluation of the white matter integrity. In this study, we evaluated optic nerve impairment in patients with neuromyelitis optica (NMO) using DTI.

**Methodology/Principal Findings:**

Optic nerve DTI were performed on 28 NMO patients and 38 normal controls. Fractional anisotropy (FA) values were measured in the anterior, middle, and posterior parts of the intraorbital optic nerve segment. For the posterior intraorbital optic nerve, FA values of BI (0.20±0.07), MI (0.24±0.16), and NA (0.25±0.14) decreased significantly compared with that of NC (0.43±0.07) (*P*<0.05), and ROC analysis demonstrated that the area under the curve (AUC) measurements for BI vs. NC, MI vs. NC, NA vs. NC, and NMO (including BI, MI, and NA) vs. NC were 0.99, 0.93, 0.88, and 0.96, respectively. The corresponding diagnostic sensitivities of ROC analysis were 100%, 80%, 80%, and 91%; and the specificities were 93%, 97%, 91%, and 93%.

**Conclusions/Significance:**

Decreased FA value in the intraorbital optic nerve, especially in the posterior part of the nerve, was demonstrated as a characteristic MR feature for NMO-related optic nerve impairment.

## Introduction

Neuromyelitis optica (NMO), also known as Devic's disease, is a primary inflammatory demyelination disease of the central nervous system that is characterized by the simultaneous or successive presentation of optic neuritis and myelitis [1]. Current knowledge shows that NMO has special clinical, laboratory, immunological, and pathological characteristics that distinguish it from multiple sclerosis [2,3]. NMO is diagnosed according to the revised Wingerchuk criteria [4] and has a relatively poor prognosis. In clinical practice, visual evaluations of NMO patients mainly include routine ophthalmological examination, visual evoked potential (VEP) recording [5], and magnetic resonance imaging (MRI). Routine ophthalmologic examination (e.g., fundoscopy) can only reveal abnormalities of the fundus oculi and optic nerve head [6]. VEP can reflect the electrophysiological changes to the functional integrity of the visual system [5], but it cannot accurately pinpoint the location of the optic nerve abnormality.

As an advanced imaging tool, MRI can be used to assess the signal of the entire visual pathway and the morphology of the visual cortex. Conventional MRI, however, can only demonstrate evident changes of the visual pathway, and it is not applicable to quantitative evaluation of the optic nerve. Among numerous advanced MR techniques, diffusion tensor imaging (DTI) is an optimal technique for the quantitative assessment of the white matter integrity and thus is suitable for the evaluation of the optic nerve. DTI parameters commonly include fractional anisotropy (FA) and mean diffusivity, and FA value reflects the degree of alignment of cellular structures and their structural integrity [7]. So far, DTI has been widely applied for the quantitative evaluation in many types of brain diseases, including amyotrophic lateral sclerosis [8], schizophrenia [9], Parkinson's disease [10], NMO [11], and multiple sclerosis [12], and it has also been used in the quantitative evaluation of the optic nerves in normal brain [13], inflamed brain [14], idiopathic demyelinating optic neuritis [15], and indirect traumatic optic neuropathy [16]. Those studies reported the FA changes in the optic nerve on some specific slices using coronal DTI, which could not reflect the FA changes along the whole intraorbital optic nerve (ION), resulting in possible bias in the assessment. Moreover, the posterior segment of ION was more frequently involved in NMO patients [17], necessitating the methodology to improve and multi-segmented FA value assessment would be more appropriate for the optic nerve impairment in NMO.

In this study, DTI was used to assess the optic nerve impairment in patients with NMO. Decreased FA values were expected in the ION, especially in the posterior part, and the decreased FA levels may be related to disease duration or VEP changes. The patient groups were classified into 3 subgroups: (1) biocular impairment (BI) group with bilateral impaired vision and abnormal VEP results; (2) monocular impairment (MI) group with unilateral impaired vision and abnormal VEP results; and (3) normal-appearing (NA) group without impaired vision and normal or abnormal VEP results based on the visual impairment. The optic DTI was performed with the patients and the normal controls, and the statistical analysis was applied to assess the optic nerve impairment in NMO patients.

## Materials and Methods

### Ethics statement

This research was approved by the Ethics Committee of the Chinese PLA General Hospital. Written informed consent was obtained from the participants, the next of kin, caretakers, or guardians on behalf of the minors/children according to the approval of the ethics committee of the local institutional review board.

### Subjects

Twenty-eight consecutive NMO patients (4 males and 24 females, ages ranging from 11 to 66 years with a mean of 35.4±13.7 years; disease duration ranged from 0.1 to 20 years with a mean of 4.3 years) were recruited from the outpatient clinic of Department of Neurology at our hospital. All the patients were diagnosed with definite NMO according to the revised Wingerchuk diagnostic criteria [4]. Of the 28 NMO patients, 18 patients (4 males and 14 females, ages ranging from 11 to 66 years with a mean of 35.1±14.3 years) suffered from BI, and 10 patients (10 females, ages ranging from 18 to 57 years with a mean of 35.9±13.2 years) suffered from MI. There were 36 optic nerves (18 cases) in BI group, 10 nerves (10 cases) in MI group, and 10 nerves (10 cases) in NA group. All the patients received an expanded disability status scale (EDSS) [18] evaluation and VEP examination. All the patients showed positive aquaporin 4 (AQP4) test.

Thirty-eight normal controls (NC) (12 males and 26 females, ages ranging from 22 to 64 years with a mean of 36.9±13.9 years) were recruited after meeting the following criteria: (1) normal ophthalmologic examination; (2) no cerebral infarction or malacia, no cranium trauma history, and no inflammatory disease of the central nervous system; (3) no history of psychoactive drug or hormone usage; and (4) normal finding on conventional MRI of the brain, especially for the visual pathway region. There was no differences of the age and gender between NMO and NC groups.

### Data acquisition

Images were acquired on a 1.5 T MR system (Signa HDxt, General Electric Healthcare, Milwaukee, WI, USA) and an eight-channel phased array head coil was used. The standard brain MR imaging protocols, including axial T2-weighted imaging (T2WI) (TR/TE = 5100 ms/113.3 ms, slice thickness = 5.0 mm, slice gap = 1.0 mm, Matrix = 320×320, FOV = 24 cm×24 cm, NEX = 2), axial T1-FLAIR (TR/TE = 1792.3 ms/24.4 ms, slice thickness = 5.0 mm, slice gap = 1.0 mm, Matrix = 228×224, FOV = 24 cm×24 cm, NEX = 2), and axial diffusion weighted imaging (DWI) (TR/TE = 4800 ms/81.8 ms, b value = 0 and 1000 s/mm^2^, slice thickness = 5.0 mm, slice gap = 1.0 mm, Matrix = 128×128, FOV = 24 cm×24 cm, NEX = 2), were performed by the same operator. All the axial data were acquired on an oblique axial plane parallel to the anterior commissure-posterior commissure (AC-PC) line.

The standard orbit MR imaging protocols, including oblique axial T2WI (TR/TE = 4100 ms/84 ms, slice thickness = 1.5 mm, slice gap = 1.0 mm, Matrix = 288×192, FOV = 18 cm×18 cm, NEX = 2) with fat saturation and oblique coronal T2WI (TR/TE = 3567 ms/85 ms, slice thickness = 3.0 mm, slice gap = 1.0 mm, Matrix = 288×224, FOV = 18 cm×18 cm, NEX = 2) with fat saturation, were performed. The oblique axial T2WI was acquired through the bilateral intraorbital segment of the optic nerve for the purpose of DTI localization. Spin-echo DTI scanning (TR/TE = 6000 ms/89.7 ms, b value = 0 and 600 s/mm^2^, NEX = 16, slice thickness = 3.0 mm, slice gap = 1.0 mm, Matrix = 128×32, FOV = 22 cm×22 cm) was performed perpendicular to the optic nerve in six non-collinear directions, and the scanning range extended from bilateral eyeballs to bilateral optic tracts. The scanning protocols were identical for all subjects.

### Signal evaluation of the optic nerve on conventional orbit MR imaging

The signal evaluation of the optic nerve was based on the comparison with the signal of the cerebral white matter on orbit MR T2WI. If the signal of optic nerve was higher than that of the cerebral white matter, it was considered as high signal, and if the signal of optic nerve was equal to that of the cerebral white matter, it was considered as iso-signal (normal). The signal evaluation was performed by visual assessment.

### Data processing

**Fig 1:**
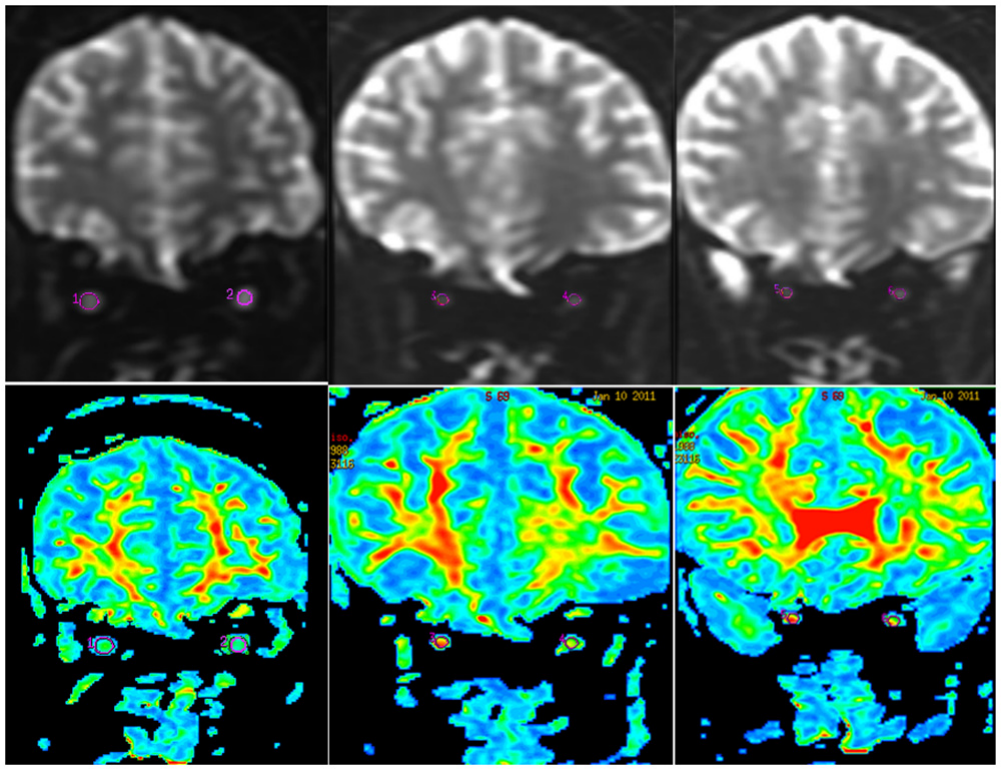
ROIs were placed on B0 images (top line) and FA maps (bottom line) at the anterior (ROI 1 and 2), middle (ROI 3 and 4) and posterior (ROI 5 and 6) segment of optic nerves (green fibers on color-coded structure images) (from left to right).

All the conventional images were reviewed on a picture archiving and communication system (PACS) workstation, and DTI data were analyzed on an Advantage Windows workstation (Functool version 9.3.01, General Electric, Milwaukee, USA) by two observers who were blind to the clinical details with consensus reading. The post-processing of DTI data was conducted as follows: (1) geometric distortion correction; (2) calculation of eigenvalues (λ1, λ2, and λ3) using single linear exponential decay; (3) derivation of FA maps by the following equation:

$$FA=\sqrt{\frac{{\left(\lambda_{1}-\lambda_{2}\right)}^{2}+{\left(\lambda_{1}-\lambda_{3}\right)}^{2}+{\left(\lambda_{2}-\lambda_{3}\right)}^{2}}{2(\lambda_{1}^{2}+\lambda_{2}^{2}+\lambda_{3}^{2})}}$$

and (4) simultaneous generation of FA map and color-coded structural map using the optimal color coding method. Regions of interest (ROIs) on the optic nerve were defined on the FA map. ION was equally divided into three parts, including anterior, middle, and posterior. ROI measurement was acquired in the middle of each part, avoiding adjacent soft tissue (Fig 1). ROI ranged from 5 mm^2^ to 9 mm^2^. FA value was measured three times and the mean FA value was calculated for each part on the same person. FA values of the anterior, middle, and posterior portions and of the whole ION were recorded.

### Statistical analysis

The descriptive data were presented as the mean±standard deviation. Homogeneity of variance was tested, and one-way analysis of variance (ANOVA) was applied using the LSD method for post-hoc multiple comparisons with variance homogeneity. Welch’s test was applied with variance nonhomogeneity, and one-way ANOVA was applied with Tamhane's T2 method for post-hoc multiple comparisons. In order to evaluate the diagnostic efficacy of FA value for optic nerve impairment, receiver operating characteristic (ROC) curves were created for comparisons among the subgroups. Pearson’s correlation analysis was then performed between the FA values and the clinical variables, including disease duration, EDSS score, the latency of P100 (positive peak at around 100 milliseconds), and the amplitude of P100. Significant difference was set at a *P* value of < 0.05. The statistical analysis was performed using SPSS 19.0.

## Results

### Observation of optic nerve on conventional orbit MR imaging

Thirty intraorbital segments of optic nerves (30/56, 54%) showed high signals and 26 segments (26/56, 46%) showed normal signals on T2WI. Of the 30 intraorbital segments, the distribution of the high signals was as follows: 5 in whole IONs (5/30), 9 in both middle and posterior IONs (9/30), and 16 only in posterior IONs (16/30), respectively. The posterior part was involved in all 30 IONs.

### Comparison of FA values among NMO subgroups and normal control

**Fig 2:**
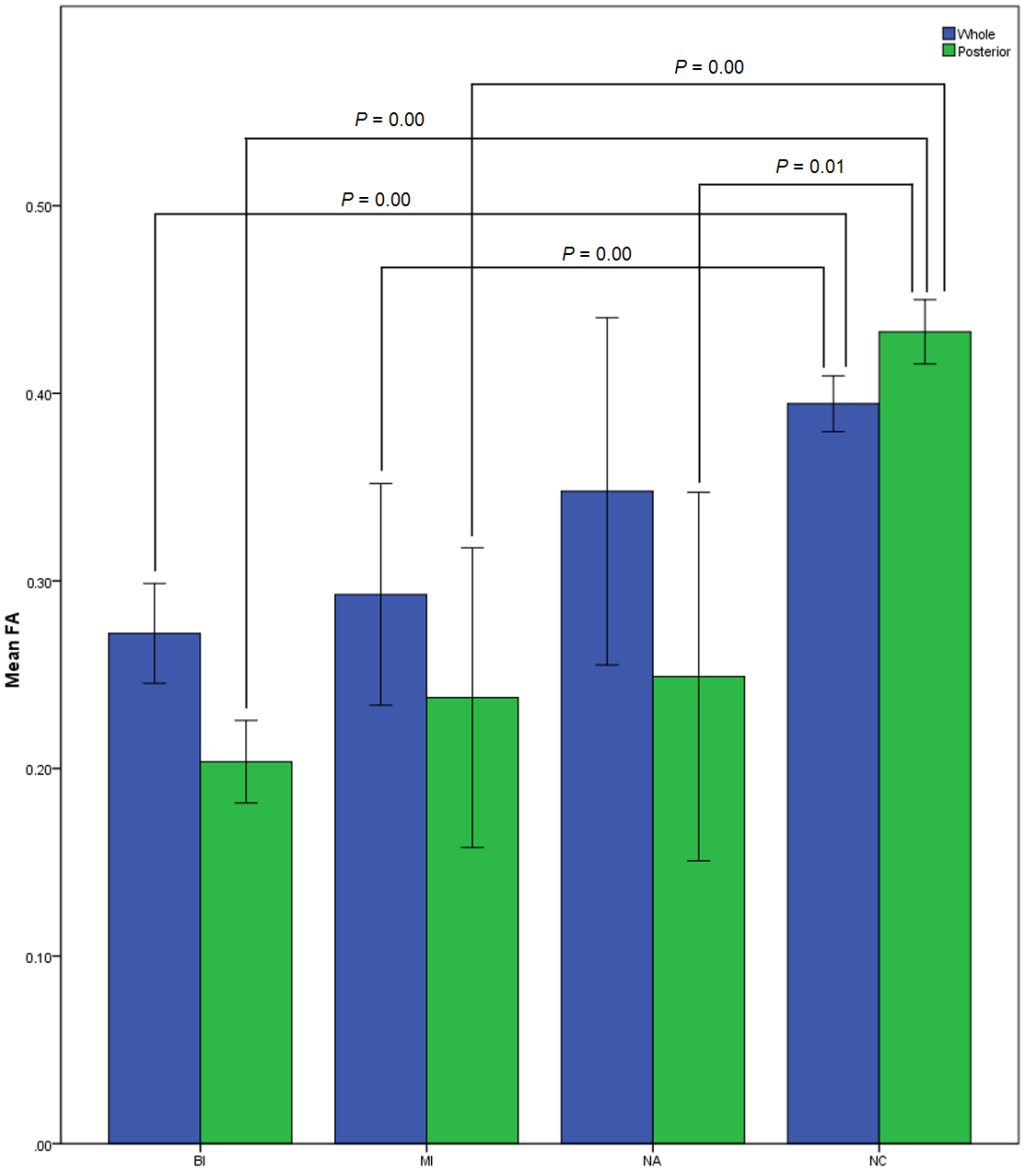
Mean FA values of the whole and posterior intraorbital optic nerve in the NMO subgroups and NC. (BI, biocular impairment; MI, monocular impairment; NA, normal-appearing; NC, normal control; whole, whole intraorbital optic nerve; posterior, posterior intraorbital optic nerve).

The mean FA values of whole ION were 0.27±0.08 in BI (including 36 optic nerves), 0.29±0.08 in MI (including 10 optic nerves), 0.35±0.13 in NA (including 10 optic nerves), and 0.39±0.07 in NC (including 76 optic nerves). ANOVA analysis demonstrated that the FA values of BI and MI decreased significantly compared with that of NC (*P* < 0.05) (Tables 1 and 2, and Fig 2).

**Table 1:** Comparisons of FA values in three segments and the whole intraorbital optic nerve among the NMO subgroups and normal controls.

	BI	MI	NA	NC	*F* value	*P* value
anterior ION	0.30±0.09	0.37±0.06	0.39±0.15	0.34±0.07	2.79	0.06
middle ION	0.31±0.12	0.27±0.09	0.40±0.14	0.45±0.07	21.09	0.00
posterior ION	0.20±0.07	0.24±0.16	0.25±0.14	0.43±0.07	22.36	0.00
whole ION	0.27±0.08	0.29±0.08	0.35±0.13	0.39±0.07	22.40	0.00

BI, biocular impairment; MI, monocular impairment; NA, normal-appearing; NC, normal control; ION, intraorbital optic nerve.

**Table 2:** Comparisons of variations in FA values among the NMO subgroups and normal controls in the whole and the posterior intraorbital optic nerve.

	*P* value	
	whole ION	posterior ION
BI vs. NC	0.00	0.00
MI vs. NC	0.02	0.00
NA vs. NC	0.87	0.01
BI vs. MI	0.98	0.94
MI vs. NA	0.85	1.00
BI vs. NA	0.49	0.91

BI, biocular impairment; MI, monocular impairment; NA, normal-appearing; NC, normal control; ION, intraorbital optic nerve.

The mean FA value of posterior ION was 0.20±0.07 in BI, 0.24±0.16 in MI, 0.25±0.14 in NA, and 0.43±0.07 in NC. ANOVA analysis demonstrated that the FA values of BI, MI, and NA decreased significantly compared with that of NC (*P* < 0.05) (Tables 1 and 2, and Fig 2).

### ROC analysis among NMO subgroups and normal control

**Fig 3:**
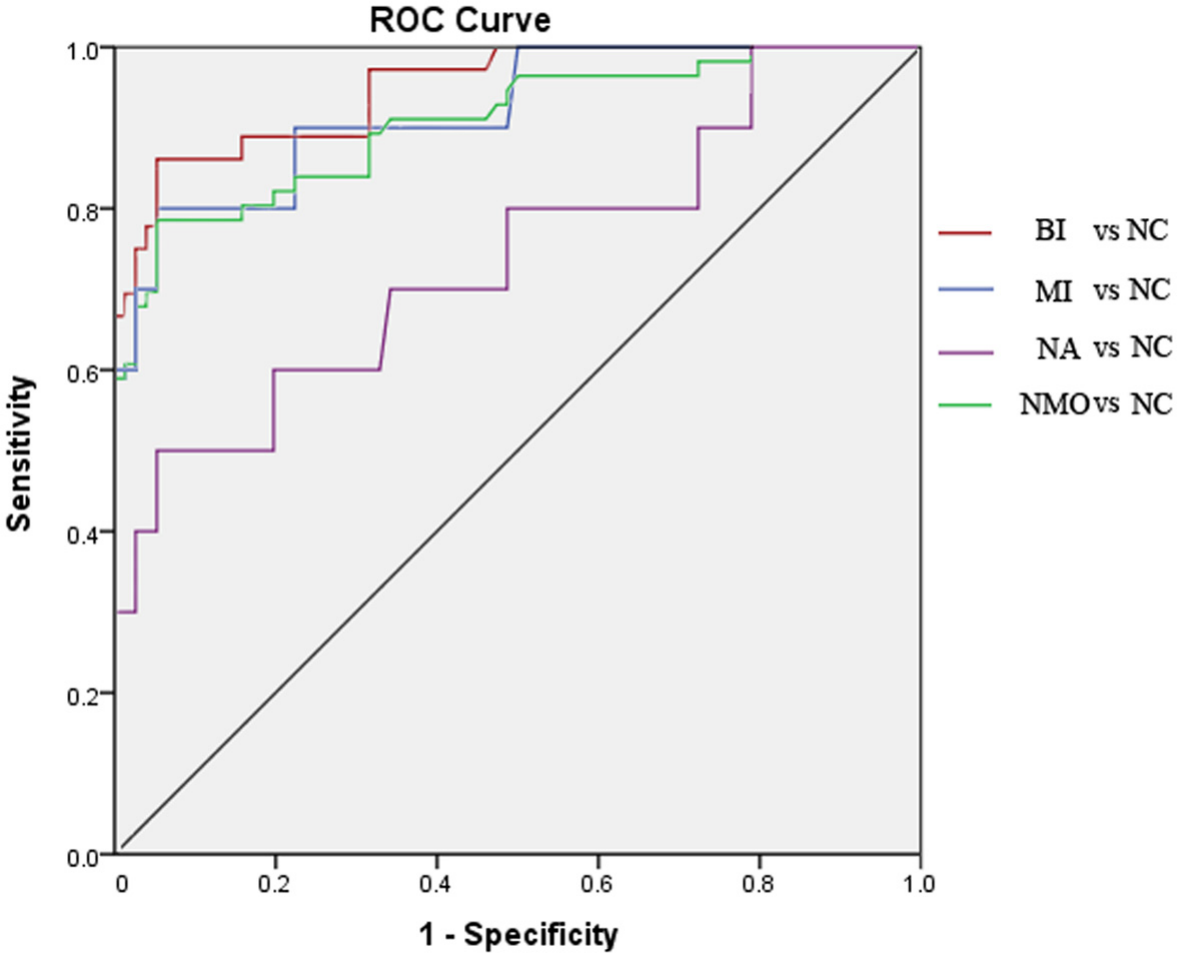
ROC curves of comparisons of FA values for the whole intraorbital optic nerve among the NMO subgroups and normal controls. The AUC (0.95) and sensitivity (86%) in BI vs. NC were larger than those in the other subgroup comparisons. (BI, biocular impairment; MI, monocular impairment; NA, normal-appearing; NC, normal control; AUC, area under the curve).

**Fig 4:**
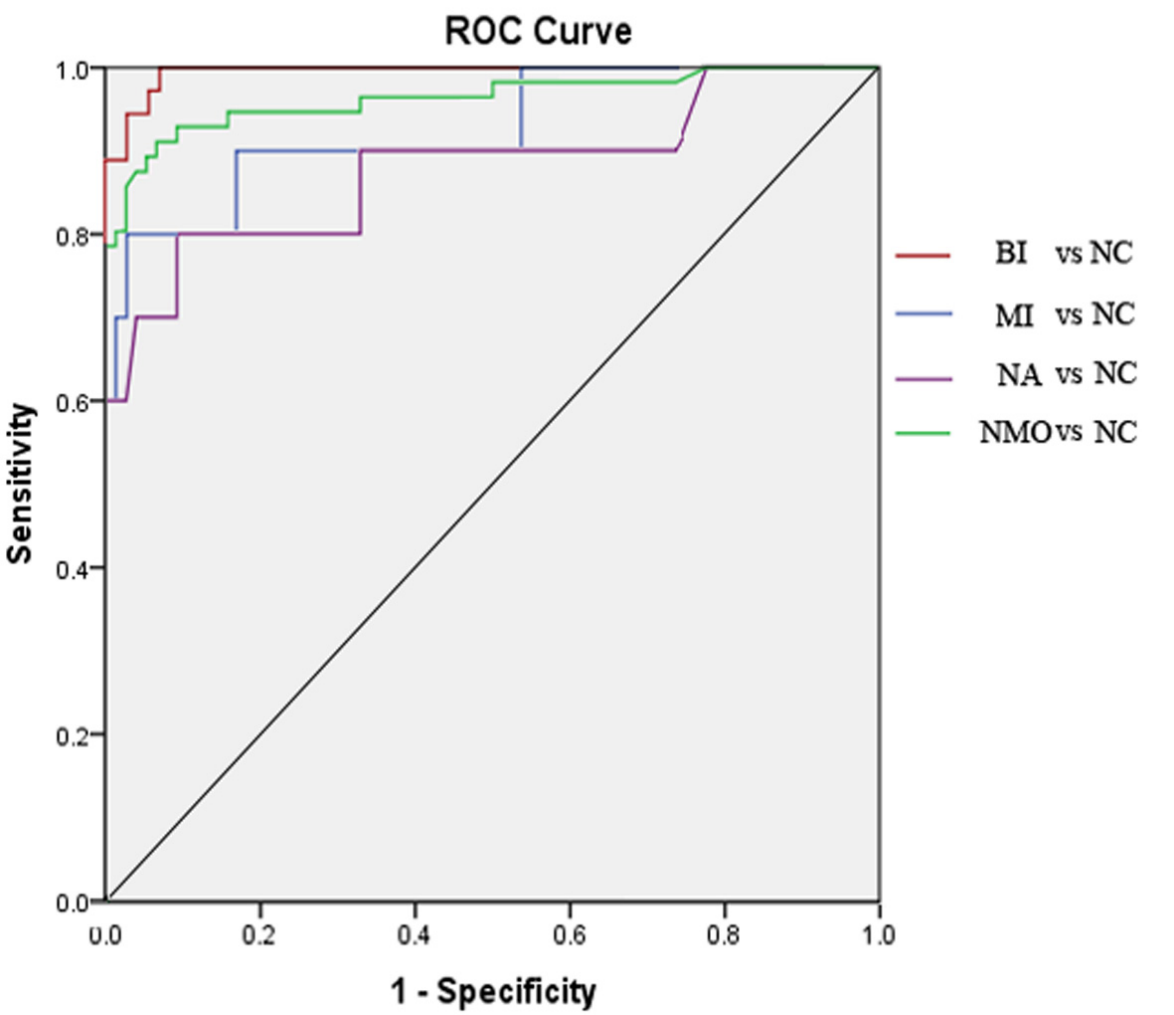
ROC curves of comparisons of FA values of the posterior intraorbital optic nerve among the NMO subgroups and normal controls. The AUC (0.99) and sensitivity (100%) in BI vs. NC were larger than those in the other subgroup comparisons. (BI, biocular impairment; MI, monocular impairment; NA, normal-appearing; NC, normal control; AUC, area under the curve).

For comparisons of FA values in BI vs. NC, MI vs. NC, NA vs. NC, and NMO (including BI, MI, and NA) vs. NC, ROC analysis revealed that the AUCs (0.99, 0.93, 0.88, and 0.96) of the posterior ION were larger than the corresponding AUCs (0.95, 0.92, 0.74, and 0.91) of the whole ION. The highest diagnostic sensitivity was demonstrated in BI vs. NC (100%), and the highest specificity in MI vs. NC (97%) for comparisons of FA values of the posterior ION (Table 3, Figs 3 and 4).

**Table 3:** ROC analysis of FA comparisons among the NMO subgroups and normal controls.

	Cut-off value	AUC	Sensitivity	Specificity
BI vs. NC				
whole ION	0.35	0.95	86%	95%
posterior ION	0.33	0.99	100%	93%
MI vs. NC				
whole ION	0.34	0.92	80%	95%
posterior ION	0.30	0.93	80%	97%
NA vs. NC				
whole ION	0.35	0.74	50%	95%
posterior ION	0.34	0.88	80%	91%
NMO vs. NC				
whole ION	0.35	0.91	79%	95%
posterior ION	0.33	0.96	91%	93%

BI, biocular impairment; MI, monocular impairment; NA, normal-appearing; NC, normal control; AUC, area under the curve; NMO including BI, MI, and NA; ION, intraorbital optic nerve.

### Correlation analysis between FA values and clinical variables

A negative correlation was found between FA value and disease duration in BI for both the whole ION (r = -0.37, *P* = 0.03) and the posterior ION (r = -0.41, *P* = 0.01), but there was no significant correlation between FA value and disease duration in MI and NA (Table 4). No correlation was shown between FA value and EDSS score in any of the three NMO subgroups (*P* > 0.05).

**Table 4:** Correlation analysis between FA value and disease duration.

	whole ION		posterior ION	
	r	*P* value	r	*P* value
BI	-0.37	0.03	-0.41	0.01
MI	-0.51	0.14	-0.51	0.14
NA	-0.28	0.43	-0.05	0.88

BI, biocular impairment; MI, monocular impairment; NA, normal-appearing; SD, standard deviation; ION, intraorbital optic nerve.

Table 5 shows that FA value negatively correlated with the latency of P100 in BI and MI for the whole ION (BI: r = -0.55, *P* = 0.00; MI: r = -0.51, *P* = 0.00) and for the posterior ION (BI: r = -0.52, *P* = 0.00; MI: r = -0.72, *P* = 0.02). FA value had a positive correlation with the amplitude of P100 in BI for the whole ION (r = 0.49, *P* = 0.00) and for the posterior ION (r = 0.40, *P* = 0.02), and in MI for the whole ION (r = 0.44, *P* = 0.00).

**Table 5:** Correlation analysis between FA value and the latency and amplitude of P100.

		latency of P100			amplitude of P100		
		mean±SD (ms)	r	*P* value	mean±SD (mv)	r	*P* value
BI							
	whole ION	121.8±9.1	-0.55	0.00	6.7±2.5	0.49	0.00
	posterior ION	121.8±9.1	-0.52	0.00	6.7±2.5	0.40	0.02
MI							
	whole ION	116.6±6.1	-0.51	0.00	6.3±1.5	0.44	0.00
	posterior ION	116.6±6.1	-0.72	0.02	6.3±1.5	0.3543	0.32
NA							
	whole ION	95.2±6.0	-0.46	0.18	10.6±4.2	0.16	0.67
	posterior ION	95.2±6.0	-0.58	0.08	10.6±4.2	-0.08	0.82

BI, biocular impairment; MI, monocular impairment; NA, normal-appearing; SD, standard deviation; ION, intraorbital optic nerve; ms, millisecond; mv, millivolt.

## Discussion

DTI is a functional MR imaging technique for studying the diffusion characteristics of water molecules, and it has been widely applied to the quantitative evaluation of white matter changes in various brain disorders [19,20,21], white matter pathways [22], cortical spinal tract [23], and cognitive [24] and spinal disorders [25,26]. Previous DTI studies [11] demonstrated abnormal changes in the normal-appearing white matter of the brain in NMO patients. In this study, DTI was used for the quantitative evaluation of the IONs in NMO patients.

NMO is a primary CNS inflammatory disease that is characterized by optic neuritis and myelitis. The evaluation of the optic nerve commonly includes the examination of visual acuity, examination of optic nerve head, and the monitoring of electrophysiological changes based on VEP. Although these examinations can assess optic nerve impairment to some extent, they cannot identify the exact nerve segment that is impaired, but this has been made possible when DTI was introduced into clinical practice.

In this study, abnormal signal changes (hyperintensity on T2WI) in the ION were detected in only 54% of the patients on conventional MRI, whereas decreased FA values were observed in 100% of the patients in all subgroups using the DTI technique. This study also demonstrated that FA values decreased significantly with increased severity of optic nerve impairment, especially for biocular impairment, which suggested that FA value can be a more sensitive clinical parameter for the evaluation of optic nerve impairment. Decreased FA value indicated restricted diffusion in the optic nerve, which may be associated with changes in cellular structure alignment and integrity in the optic nerve [7]. ROC analysis showed that the diagnostic value of FA measurement was relatively high for optic nerve impairment in NMO patients, especially in those with BI and MI, as demonstrated by the high sensitivity and specificity. FA value may play a key roles in the diagnosis and evaluation of NMO patients. Therefore, our data suggested that optic DTI examination should be performed at the time of disease onset.

Conventional MRI demonstrated that the posterior ION had a higher propensity for impairment, although the specific mechanisms have not been elucidated so far [17]. Our study demonstrated that the FA value for posterior ION was lower in NA than in NC (*P* < 0.05), while there was no significant difference between NA and NC for the whole ION (*P* > 0.05), which suggested that FA measurement in posterior ION could reveal concealed impairment before visual acuity decreased. Our study also demonstrated that the AUC of the posterior ION was larger than that of the whole ION in each subgroup comparison, which suggested that the diagnostic value of FA measurement was higher in the posterior ION than in the whole ION. For the BI and MI patients, the FA values of the whole and posterior IONs had similar diagnostic value in detecting optic nerve impairment. For the NA patients, however, the FA value of the whole ION had relatively limited diagnostic value based on the relatively small AUC (0.74) and low sensitivity (50%), while the FA value of the posterior ION performed better diagnostically with a larger AUC (0.88) and a higher sensitivity (80%). Therefore, the diagnostic efficacy of FA measurement of posterior ION may be better than that of whole ION.

Our study showed that there was no significant difference in FA values in the comparisons of BI vs. MI, MI vs. NA, and BI vs. NA, which indicated that FA value had limited efficacy in differentiating NMO subgroups.

Additional analysis revealed that the FA values did not correlate with the EDSS scores, which may be due to the multiple parameters that made up the EDSS scores, including functional evaluations of visual system, brain stem, pyramidal tract, cerebellum, sensory system, and bladder/rectum. In this study, the FA values were only measured for the optic nerves, which may explain the lack of correlation between FA and EDSS scores. Table 4 shows that the FA values of the BI subgroup negatively correlated with disease duration for both the whole ION (r = -0.37, *P* = 0.03) and the posterior ION (r = -0.41, *P* = 0.01), which suggested that FA value may reflect the severity of optic nerve impairment and that the FA value of posterior ION might be a good parameter for the longitudinal evaluation of optic nerve impairment in NMO patients.

As an electrophysiological technique, VEP measurement was widely used in the clinical practice for the functional evaluation of visual pathways, including analysis of P100 latency and amplitude. Abnormal VEP readings mainly included the prolongation of P100 latency and the decreased amplitude of P100. In this study, a negative correlation between FA value and P100 latency and a positive correlation between FA value and P100 amplitude were observed in BI and MI. Therefore, FA value can be used to indicate visual damage and abnormal VEP in BI and MI patients.

As a preliminary quantitative evaluation of optic nerve using the DTI technique in NMO patients, several limitations existed in this study. First, this was an *in vivo* MRI study without pathological evidence. Second, this was a cross-sectional study, and longitudinal evaluation and validation for the clinical use of FA values are needed, especially for the usefulness of FA measurement in diagnosing NMO with normal-appearing optic nerves. Third, Only NMO patients were observed in this study although several demyelinating diseasess, such as MS and optic neuritis, could involve the optic nerve, and then the differential diagnosis may be wanting with these disorders.

In conclusion, optic nerve impairment was assessed in NMO patients based on FA measurement using DTI. Decreased FA value in the ION, especially in the posterior segment, was found to be associated with NMO-related optic nerve impairment. Therefore, DTI may be a simple and effective tool for the evaluation of optic nerves in NMO patients.
